# Cardiac Amyloidosis: A Diagnostic Dilemma and the Role of a Novel Electrocardiogram Criterion

**DOI:** 10.7759/cureus.26561

**Published:** 2022-07-04

**Authors:** Muhammad Asad Hanif, Tuoyo Omasan Mene-Afejuku, Olivera Chandler, Julian Diaz Fraga

**Affiliations:** 1 Department of Internal Medicine, Tower Health, Reading Hospital, West Reading, USA; 2 Department of Cardiology, Tower Health, Reading Hospital, West Reading, USA

**Keywords:** plasma cell myeloma, left ventricular hypertrophy, syncope, heart failure, cardiac amyloidosis

## Abstract

Cardiac amyloidosis is an infiltrative disease of the myocardium. Nearly all cases of clinical cardiac amyloidosis are caused by transthyretin amyloidosis or light chain amyloidosis. Clinical manifestations are consistent with those of refractory heart failure secondary to irreversible restrictive cardiomyopathy, autonomic abnormalities as well as neuropathy. Delay in diagnosis is a challenge, as symptoms and signs of cardiac amyloidosis are nonspecific. One of the hallmarks of cardiac amyloidosis is the discordance between the increased left ventricular wall thickness and low QRS voltages on the electrocardiogram. Diagnostic delay may lead to deleterious consequences as prompt therapy, if feasible, would be hampered.

We, therefore, present a case of cardiac amyloidosis presenting with syncope and refractory heart failure to highlight the diagnostic dilemma as well as to stress upon the utility of a novel electrocardiogram criterion that may assist in the diagnosis of cardiac amyloidosis.

## Introduction

Cardiac amyloidosis is a diagnosis that is frequently missed or delayed [1]. The true incidence and prevalence vary widely because of the diagnostic difficulties that are characteristic of this disease entity [1,2]. The prognosis of cardiac amyloidosis depends on the type [1-3]. Late detection may result in a delay in therapy and an unfavorable prognosis [2]. Patients with cardiac amyloidosis may present with features of refractory heart failure (HF), which can also be associated with symptoms of autonomic neuropathy such as syncope [3,4]. As the disease generally presents with nonspecific symptoms [5], one must have a high index of suspicion to diagnose it. To highlight this, we present a case of syncope with an exacerbation of HF with preserved ejection fraction (HFpEF) that was subsequently diagnosed with cardiac amyloid light (AL) chain amyloidosis. We also shed light upon the utility of a novel electrocardiogram criterion that can assist in the diagnosis of cardiac amyloidosis in men with a bundle branch block.

## Case presentation

A 70-year-old male with a history of essential hypertension, type 2 diabetes mellitus, coronary artery disease with coronary artery bypass grafts, aortic stenosis with bioprosthetic aortic valve replacement, HFpEF, severe pulmonary hypertension, atrial fibrillation, and tachy-Brady syndrome with pacemaker placement was brought in by the emergency medical service (EMS), after an episode of syncope. The episode was preceded by lightheadedness, shortness of breath, and fatigue. The syncope was witnessed by his wife who reported that the patient was unconscious for about 5-10 minutes and had labored breathing during that time. There were no reports of shaking, tongue biting, urinary or fecal incontinence. The patient denied any palpitations before or after the event. He had been having intermittent episodes of lightheadedness and weakness for the past several weeks. The patient also reported weight loss which he attributed to the use of semaglutide. He was afebrile on presentation with blood pressure of 122/71 mmHg, heart rate of 77 beats per minute, respiratory rate of 14 breaths per minute, and oxygen saturation of 98% on room air. His physical exam was significant for a body mass index of 36 kg/m^2^, elevated jugular venous pressure (JVP), bibasilar lung crackles, systolic murmur at the left sternal border with inspiratory accentuation, and bilateral lower extremity pitting edema. Heart rhythm was irregularly irregular. Orthostatic vitals were normal.

He had a troponin I of 0.19 ng/mL (reference range: ≤0.06 ng/mL) which subsequently plateaued around 0.4-0.5 ng/mL. His brain natiuretic peptide (BNP) was 781 pg/mL (reference range: 0-100 pg/mL), which was close to his baseline. Initial laboratory values are shown in Table 1.

**Table 1:** Admission laboratory values. WBC = white blood cell, BUN= blood urea nitrogen, BNP= brain natriuretic peptide

Laboratory test	On admission	Lab reference range
WBC, 10E3/µL	9.0	4.8-10.8
Hemoglobin, g/dL	14.6	14.0-17.5
Platelets, 10E3/µL	159	130-400
Sodium, mmol/L	137	136-145
Potassium, mmol/L	3.4	3.5-5.1
Chloride, mmol/L	99	98-107
Bicarbonate, mmol/L	28.4	21.0-31.0
Glucose, mg/dL	124	70-99
BUN, mg/dL	32	7-25
Creatinine, mg/dL	1.07	0.60-1.30
Calcium, mg/dL	9.4	8.6-10.3
Albumin, g/dL	3.4	3.5-5.7
Total protein, g/dL	6.9	6.4-8.9
Total Bilirubin, mg/dL	0.9	0.3-1.0
Direct Bilirubin, mg/dL	0.2	0.0-0.2
Aspartate aminotransferase, IU/L	40	13-39
Alanine aminotransferase, IU/L	23	7-52
BNP, pg/mL	781	0-100
Troponin I, ng/mL	0.19	<=0.06
Magnesium, mg/dL	2.0	1.9-2.7

**Figure 1:**
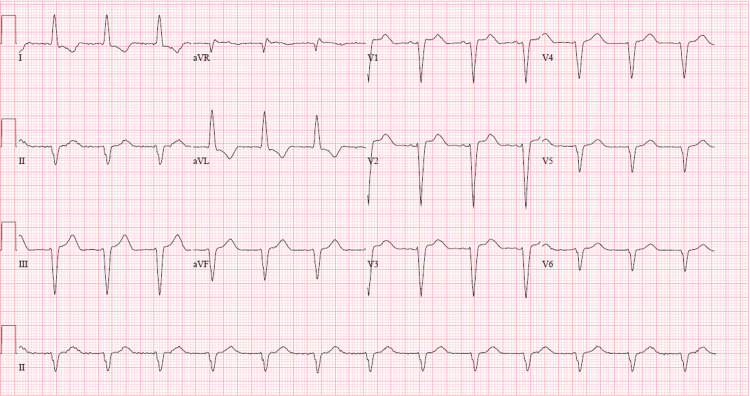
Electrocardiogram shows normal sinus rhythm and intraventricular conduction delay of left bundle branch block morphology.

**Figure 2:**
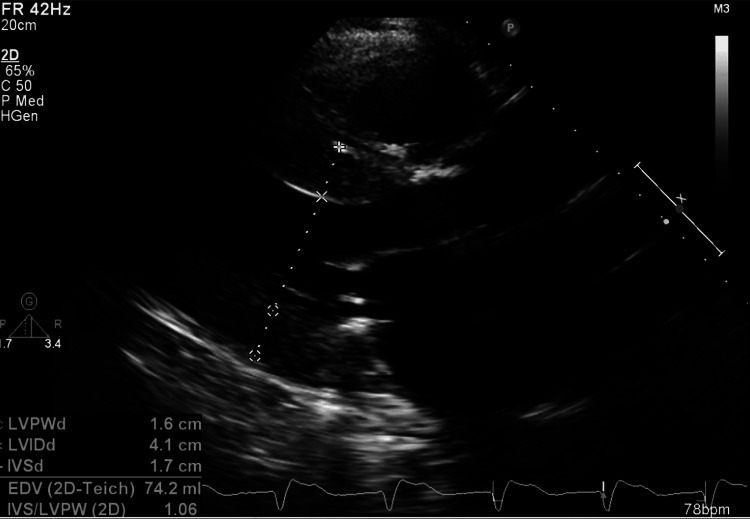
Transthoracic echocardiogram (Parasternal long-axis view) showing left ventricular hypertrophy and dilated left atrium.

Electrocardiogram (EKG, Figure 1) showed normal sinus rhythm, left axis deviation, and a left bundle branch block, and was unchanged from prior EKGs. Computerized tomographic angiography of the chest was negative for acute pulmonary embolism but revealed moderate cardiomegaly, moderate right-sided pleural effusion, and diffuse smooth interstitial thickening indicating fluid overload. Pacemaker interrogation showed normal pacemaker function with no pauses, atrioventricular blocks, or ventricular arrhythmias. A transthoracic echocardiogram (TTE) revealed a left ventricular ejection fraction (LVEF) of 50% (LVEF was 62% on TTE three months ago) with no new regional wall motion abnormalities, indeterminate diastolic function in the setting of atrial fibrillation and moderate to severe concentric hypertrophy of the left ventricle with elevated left ventricular end-diastolic pressure estimate by tissue Doppler imaging (as shown in Figure 2).

The stroke volume index was 24.5 mL/m^2^/beat (reference range: 33-47 mL/m^2^/beat). His most recent left heart catheterization was three months ago, which showed only mild nonobstructive disease. The patient was diuresed aggressively with a furosemide drip at 20 mg/hr for his volume overload, which resulted in resolution of his elevated JVP, bibasilar crackles, and lower extremity edema, but he continued to have dyspnea and fatigue. Due to his TTE showing moderate to severe concentric hypertrophy of the left ventricle, very low stroke volume index, and small left ventricular end-diastolic size, cardiac amyloidosis was suspected, and further workup was done.

**Figure 3:**
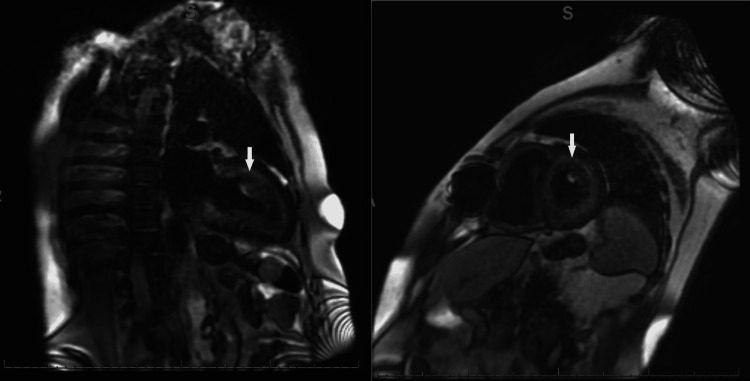
(Left, right) Gadolinium-enhanced cardiac MRI showing diffuse heterogeneous enhancement overlying the entire subendocardial circumference extending into at least 50% of the myocardial thickness (white arrows).

**Figure 4:**
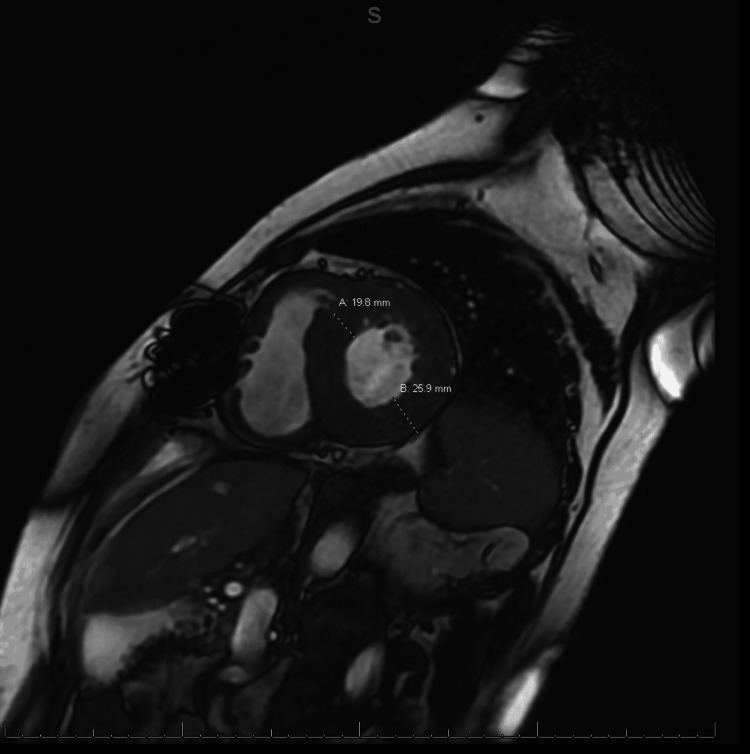
Cardiac MRI showing anteroseptal wall thickness of 19.8 mm and posterior lateral wall thickness of 25.9 mm.

**Figure 5:**
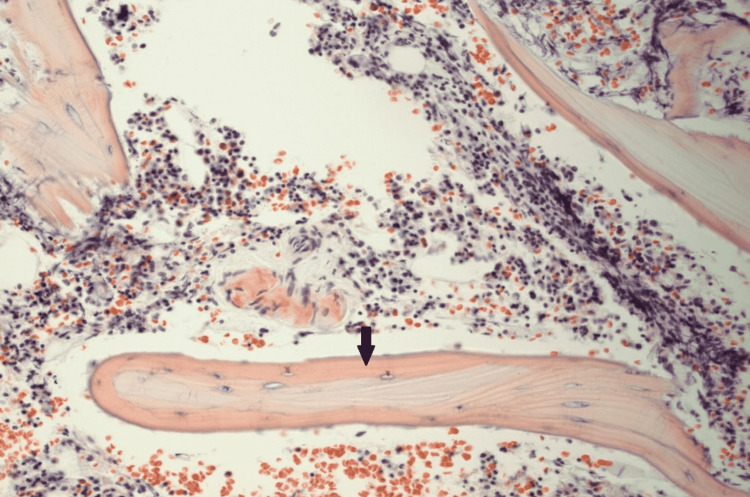
Congo red stain of the bone marrow biopsy showing amyloid deposits in the vessel walls (black arrow).

Gadolinium-enhanced cardiac magnetic resonance imaging (MRI) revealed diffuse heterogenous circumferential subendocardial enhancement throughout the left ventricle, highly suggestive of amyloidosis as shown in Figures 3, 4. Blood work revealed elevated lambda-free light chains at 245.23 mg/L (reference range: 5.71-26.3 mg/L). Subsequently, a bone marrow biopsy was performed which was positive for plasma cell myeloma with Congo red stain revealing amyloid deposition (Figure 5). Thus, a diagnosis of cardiac AL amyloidosis was made and chemotherapy with cyclophosphamide, bortezomib, and dexamethasone (CyBorD) was initiated with a plan to add daratumumab as an outpatient.

The patient’s syncope on presentation was thought to be a vagal episode due to autonomic instability from his AL amyloidosis. He was discharged to post-acute rehab with a plan to continue outpatient chemotherapy for AL amyloidosis. Unfortunately, the patient suffered a cardiac arrest at rehab eight days after discharge and was unable to be successfully resuscitated.

## Discussion

Patients with cardiac amyloidosis typically present with symptoms and signs such as dyspnea, lower extremity edema, elevated JVP, hepatic congestion, and ascites. These are caused by amyloid deposition causing restrictive cardiomyopathy [5-7]. Features of advanced disease include symptoms and signs of low cardiac output which include diminished pulse pressure and diminished capillary refill [6]. Patients can also present with syncope or presyncope, the cause of which more commonly is bradyarrhythmia or advanced atrioventricular block [7]. Postural or exertional hypotension caused by excessive diuresis or autonomic neuropathy may also lead to syncope in these patients. In our case, the patient had many of these symptoms and signs, but the EKG did not meet low voltage criteria. On the contrary, the EKG nearly met the Modified Cornell criteria for left ventricular hypertrophy as the R wave in AVL was 11mm. Thus, it took several days till a closer review of the echocardiogram findings of bi-atrial enlargement, moderate to severe left ventricular hypertrophy, and a very low stroke volume, all in keeping with restrictive cardiomyopathy, leading to cardiac amyloidosis being suspected as a potential diagnosis.

In retrospect, we believe that the use of an EKG criterion indexed to left ventricular wall thickness as described by Sharma et al. [8] to diagnose cardiac amyloidosis in men with a bundle branch block could have been used to screen our patient to help in reaching the diagnosis early. The criterion uses the total QRS score, calculated as the sum of the total QRS amplitude in all 12 EKG leads, divided by the average left ventricular wall thickness on TTE. A score of less than 92.5 is 100% sensitive and 83.3% specific for cardiac amyloidosis. Our patient had a total QRS score of 138 and an average left ventricular wall thickness of 1.65 cm on TTE. His ratio comes out to be 83.6 which points toward a diagnosis of amyloid cardiomyopathy. Hence, this score may be utilized in patients with a bundle branch block who have evidence of significant ventricular hypertrophy on TTE and refractory heart failure or syncope to screen for cardiac amyloidosis.

## Conclusions

Cardiac amyloidosis may be a masquerader of many clinical conditions and a strong clinical suspicion is necessary for early diagnosis. This becomes even more challenging in patients with a bundle branch block as they are less likely to have a classic low voltage EKG and a discrepancy between the QRS amplitude and the left ventricular wall thickness. The EKG criterion referenced in our report can be used to screen men with a bundle branch block to assist in timely diagnosis. Institution of prompt therapy may favorably alter the grim prognosis associated with cardiac amyloidosis.
